# The effects of plasma to red blood cells transfusion ratio on in-hospital mortality in patients with acute type A aortic dissection

**DOI:** 10.3389/fcvm.2023.1091468

**Published:** 2023-05-11

**Authors:** Run Yao, Danyang Yan, Xiangjie Fu, Ying Deng, Xi Xie, Ning Li

**Affiliations:** ^1^ Department of Blood Transfusion, National Clinical Research Center for Geriatric Disorders, Xiangya Hospital, Clinical Transfusion Research Center, Central South University, Changsha, China; ^2^ Office, Ningxiang People's Hospital Affiliated to Hunan University of Traditional Chinese Medicine, Ningxiang, China

**Keywords:** transfusion, mortality, acute aortic dissection, plasma, red blood cells

## Abstract

**Background:**

Blood transfusion is a frequent and necessary practice in acute type A aortic dissection (AAAD) patients, but the effect of plasma/red blood cells (RBCs) ratio on mortality remains unclear. The aim of this study is to investigate the association between plasma/RBCs transfusion ratio and in-hospital mortality in patients with AAAD.

**Methods:**

Patients were admitted to Xiangya Hospital of Central South University from January 1, 2016 to December 31, 2021. Clinical parameters were recorded. Multivariate Cox regression model was used to analyze the association between transfusion and in-hospital mortality. We used the smooth curve fitting and segmented regression model to assess the threshold effect between plasma/RBCs transfusion ratio and in-hospital mortality in patients with AAAD.

**Results:**

The volumes of RBCs [14.00 (10.12–20.50) unit] and plasma [19.25 (14.72–28.15) unit] transfused in non-survivors were significantly higher than in survivors [RBCs: 8.00 (5.50–12.00) unit]; plasma: [10.35 (6.50–15.22) unit]. Multivariate Cox regression analysis showed plasma transfusion was an independent risk factor of in-hospital mortality. Adjusted HR was 1.03 (95% CI: 0.96–1.11) for RBCs transfusion and 1.08 (95% CI: 1.03–1.13) for plasma transfusion. In the spline smoothing plot, mortality risk increased with plasma/RBCs transfusion ratio leveling up to the turning point 1. Optimal plasma/RBCs transfusion ratio with least mortality risk was 1. When the plasma/RBCs ratio was <1 (adjusted HR per 0.1 ratio: 0.28, 95% CI per 0.1 ratio: 0.17–0.45), mortality risk decreased with the increase of ratio. When the plasma/RBCs ratio was 1–1.5 (adjusted HR per 0.1 ratio: 2.73, 95% CI per 0.1 ratio:1.13–6.62), mortality risk increased rapidly with the increase of ratio. When the plasma/RBCs ratio was >1.5 (adjusted HR per 0.1 ratio: 1.09, 95% CI per 0.1 ratio:0.97–1.23), mortality risk tended to reach saturation, and increased non-significantly with the increase of ratio.

**Conclusion:**

A 1:1 plasma/RBCs ratio was associated with the lowest mortality in the patients with AAAD. And non-linear relationship existed between plasma/RBCs ratio and mortality.

## 1. Introduction

Aortic dissection is a disease with a very rapid onset and a high mortality rate. It can be divided into type A and Type B. Acute type A aortic dissection (AAAD), involves the lesions of the ascending aorta and aortic arch, are more serious and may rupture the great vessels at any time, thus leading to the loss of life (1). Surgery is the best treatment modality for AAAD, and 75.3% of AAAD patients are treated by surgery (2). Excessive bleeding is a recognized and common problem in aortic dissection surgery, which increases complications and mortality (3). Both excessive bleeding and cardiopulmonary bypass could cause the consumption of coagulation factors and haemodilution, which manifests coagulation dysfunction. Therefore, blood transfusion during operation to correct bleeding and coagulation system disorders is inevitable (4). Although blood transfusion has potential benefits, it can also lead to several blood transfusion-related complications (including cellular hypoxia, wound infections, renal dysfunction, transfusion-related immune and circulation problems), and even severe sepsis and hospital mortality (5). The effects of transfusion on mortality are controversial. Some evidence indicated that blood transfusion could increase mortality, especially in trauma and critically ill surgical patients (6, 7), whereas, other studies reported that no relationship between blood transfusion and mortality in colon and rectal cancer resection patients (8). The decision regarding when a patient requires transfusion and transfusion volume varied significantly among intensivists, anesthetists, and cardiac surgeons (9). At present, the selection and application of the blood products composition and proportion remains considerable controversial (10).

Targeting bleeding-related coagulopathy with a balanced transfusion strategy included the optimal plasma to red blood cells (RBCs) transfusion ratio which is closed to 1:1 (11). In the meta-analysis of Rahouma M et al., they evaluated the benefits of the transfusion ratio in surgical patients and found that high plasma to red blood cells transfusion ratio was beneficial and might improve survival at 24 h and 30 days (12). Nederpelt CJ et al. suggested that, compared to a 1:1 plasma/RBCs ratio, lower plasma/RBCs ratios increased incrementally the mortality in patients with traumatic hemorrhage (13). To date, the optimal plasma/RBCs transfusion ratio with AAAD patients and the impact of the plasma/RBCs transfusion ratio on mortality have not been evaluated. Therefore, the aim of this single retrospective study is to investigate the possible relationship between plasma/RBCs transfusion ratio and in-hospital mortality of patients with AAAD.

## 2. Material and methods

### 2.1. Study participants

AAAD patients underwent emergent operations at our institution between January 1, 2016 to December 31, 2021 were enrolled. The computed tomography scanning and echocardiography were used to identified the diagnosis of AAAD. All patients were admitted to the hospital from hours to several days after the onset of symptoms, and emergent surgery (within 48 h from admission) was performed after establishing the diagnosis of AAAD. Surgical patients whose age were older than 18, diagnosed with AAAD and received at least one unit RBCs during surgery (14) were included to participants. However, patients who died during operation, died within 24 h after operation (15), RBCs transfused <one unit and no plasma transfused were excluded. The study was approved by Medical Ethics Committee of the Xiangya Hospital of Central South University (ethical number: 2019010038). This research was the retrospective study, so the individual consent was waived.

### 2.2. Data collection

Demographic characteristics, disease history, preoperative laboratory indicators, and hospitalization management of all patients were collected retrospectively through the electronic medical record system. Demographic characteristics included age and gender. Disease history included smoking, drinking, Marfan syndrome, Penn class, hypertension, diabetes mellitus, hemopericardium, chronic renal failure, cerebrovascular disease and coronary artery disease. Preoperative laboratory indicators included activated partial thromboplastin time (APTT), prothrombin time (PT), international normalized ratio (INR), D-dimer, fibrin/fibrinogen degradation product (FDP), leukocyte, platelet count and hemoglobin. All laboratory data were obtained from the routine blood tests and their blood samples were collected when they were admitted to the hospital. Hospitalization management included cardiopulmonary bypass (CPB) time, aortic cross clamp time, circulatory arrest time, ventilator, autologous blood transfusion ≥500 ml, operative time and surgery type. In addition, we also recorded intensive care unit (ICU) stay of all patients.

A history of smoking was defined as at least 1 cigarette daily for more than one year or previous daily smoking (16). A history of drinking was defined as alcohol consumption at least once a week over a year or quitting for less than three years (16). Cerebrovascular disease was diagnosed by a combination of symptoms (headache), physical examination (weakness or numbness on one side of the body, difficulty speaking or understanding speech, loss of balance or coordination), imaging tests (computed tomography or magnetic resonance imaging showed the presence of blood clots, bleeding, or other abnormalities in the blood vessels or brain) (17). Coronary artery disease was diagnosed by a combination of symptoms (chest pain or angina, shortness of breath, fatigue), physical examination (auscultation of the heart or lungs), imaging tests (coronary angiography and x-rays, computed tomography or magnetic resonance imaging showed any blockages or narrowing) (18).

### 2.3. Transfusion practices

Blood products included RBCs, plasma, cryoprecipitate, and apheresis platelet that patients transfused intraoperatively and postoperatively were recorded (19). Each unit of RBCs or plasma was obtained from 200 ml whole blood. Apheresis was the collection of platelets from a donor with a blood component apheresis machine that contained at least 250 × 10^9^ platelets. Each bag of cryoprecipitate was made from 400 ml whole blood with a volume of 25 ml ± 5 ml/bag, which mainly contained ≥80 IU factor VIII, fibrinogen ≥150 mg, von Willebrand factor, fibrin, coagulation factor XIII and so on. Ten bags of cryoprecipitate were an adult therapeutic dose (20). Intraoperative blood transfusion was performed at the discretion of intensivists, cardiac surgeons and attending anesthesiologists and did not need to be based on a protocol or agreement.

### 2.4. Outcomes

In-hospital mortality was the primary end point. All patients were followed up from the date of hospital admission up to the date of in-hospital death or discharge.

### 2.5. Statistical analysis

Firstly, the data distribution between the survival and non-survival groups was compared. Mean ± standard deviation or medians (interquartile ranges) was used to express continuous variables. And the one-way ANOVA (Analysis of Variance) was used to analysis normally distributed continuous variables, the Kruskal-Wallis test was used to analysis skewed continuous variables. The normality of continuous variables was tested by the Shapiro-Francia normality test. Frequency (percentage) was used to express categorical variables. The chi-squared test was used to analysis categorical variables. Fisher's exact test was used to analysis the frequency <5 in one of the groups. There was no multicollinearity between the independent variables. The Cox proportional hazards model was utilized to verify the proportional hazards assumption, which was based on the Schoenfeld residuals. A univariate Cox regression analysis was used to evaluate the associations between the variables and in-hospital mortality. To accurately study the relationship between blood transfusion and the risk of death in AAAD patients, unadjusted and adjusted multivariate Cox regression models were used to estimate the hazard ratio (HR) and 95% confidence interval (CI) for in-hospital mortality risk. We adjusted age, gender and potential confounders in the Model III. The potential confounders were selected base on the basis of their associations with in-hospital mortality (*P-*value < 0.10) (21). Then, we used smooth curve fitting to visualize if there was a non-linear relationship between plasma/RBCs transfusion ratio and in-hospital mortality in AAAD patients with adjustment for covariates. And we used segmented regression model and LRT (likelihood ratio test) to analyze the threshold effect between in-hospital mortality and plasma/RBCs transfusion ratio.

All analyses were performed with R (http://www.R-project.org) and EmpowerStats software (www.empowerstats.com, X&Y solutions, Inc. Boston MA).

## 3. Results

### 3.1. Participants' selection

**Figure 1:**
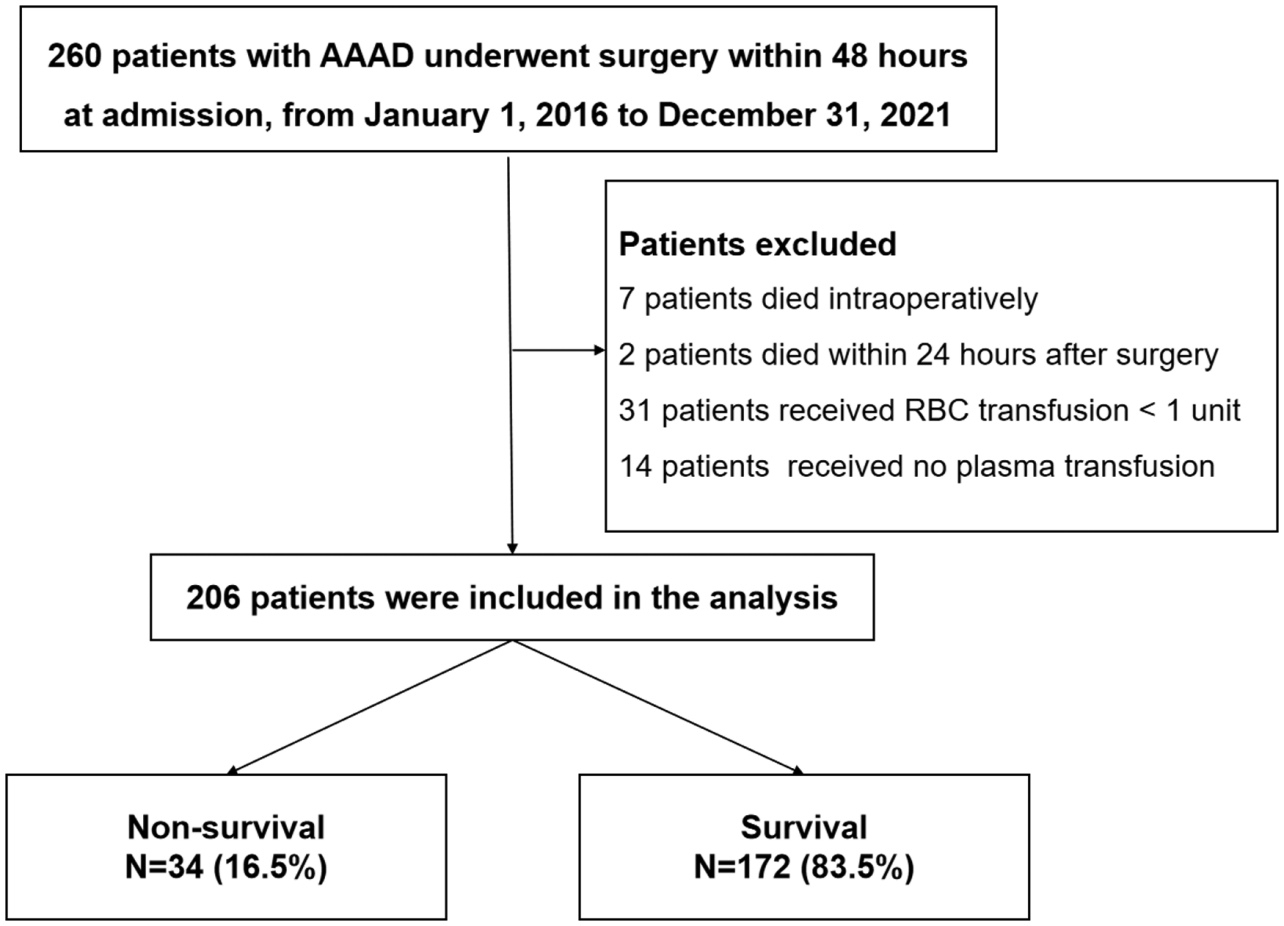
Flow chart.

A total of 260 patients' records were reviewed. Among them, seven patients died intraoperatively, two patients died within 24 h after surgery, thirty-one patients received RBCs transfusion < one unit and fourteen patients hadn’t received plasma transfusion. The remaining 206 patients were enrolled in the final analysis. The in-hospital mortality rate was 16.5% (34 patients) among these patients (Figure 1).

### 3.2. Patients' characteristics

The baseline characteristics of the cohort were shown in Table 1. There were 111 (64.53%) males in survival group and 22 (64.71%) males in non-survival group. Mean age of survival patients and non-survival patients were 50.13 ± 10.52 and 51.91 ± 11.30, respectively. A higher prevalence of cerebrovascular diseases (20.59% vs. 3.49%, *P* < 0.001) was found in the non-survival group. For the blood products transfusion in non-survival group, the volume of RBCs and Plasma were 14.00 (10.12–20.50) units and 19.25 (14.72–28.15) units, which were significantly higher than survival group (*P* < 0.001). The plasma/RBCs transfusion ratio were 1.41 ± 0.59 in survival group and 1.50 ± 0.66 in non-survival group. The patients received platelet transfusion in the non-survival group [2.00 (1.00–3.00)] was more than in the survival group [1.00 (1.00–2.00), *P* = 0.010]. ICU stay in non-survival group was longer than in survival group [9.50 (4.00–13.50) vs. 6.00 (4.00–9.00), *P* < 0.001]. Operative time in non-survival group was higher than survival group (11.74 ± 4.42 vs. 10.68 ± 3.39, *P* = 0.050). We found there were no statistically significant differences between the survivor and non-survivor groups in drinking, smoking, INR, APTT, PT, D-dimer, FDP, leukocyte, platelet count, hemoglobin, hypertension, diabetes mellitus, hemopericardium, chronic renal failure, coronary artery disease, Marfan syndrome, Penn class, CPB time, aortic cross clamp time, circulatory arrest time, ventilator, autologous blood ≥500 ml and surgery type.

**Table 1:** Demographic and clinical characteristics of all patients with AAAD.

Variables	Survival (*n* = 172)	Non-survival (*n* = 34)	*P*-value
Age (years)	50.13 ± 10.52	51.91 ± 11.30	0.375
Gender (male)	111 (64.53%)	22 (64.71%)	0.985
INR	1.15 ± 0.23	1.14 ± 0.13	0.651
APTT (s)	35.43 ± 6.70	34.59 ± 4.65	0.485
PT (s)	14.47 ± 2.04	14.50 ± 1.72	0.953
Leukocyte (10^9^/L)	11.79 ± 4.03	11.65 ± 3.93	0.855
Platelet count (10^9^/L)	171.23 ± 72.18	160.88 ± 53.66	0.429
Hemoglobin (g/L)	121.13 ± 18.61	119.68 ± 18.69	0.677
D-dimer (μg/ml)	1.99 (1.23–2.76)	2.01 (1.32–2.44)	0.757
FDP (μg/ml)	16.49 (9.07–21.32)	18.76 (12.73–23.43)	0.231
Smoking	75 (43.60%)	14 (41.18%)	0.794
Drinking	65 (37.79%)	7 (20.59%)	0.055
Marfan syndrome	12 (6.98%)	2 (5.88%)	0.817
Hypertension	121 (70.35%)	21 (61.76%)	0.323
Diabetes mellitus	11 (6.40%)	0 (0.00%)	0.130
Hemopericardium	16 (9.30%)	2 (5.88%)	0.519
Chronic renal failure	6 (3.49%)	2 (5.88%)	0.509
Cerebrovascular disease	6 (3.49%)	7 (20.59%)	<0.001
Coronary artery disease	17 (9.88%)	4 (11.76%)	0.740
Penn class			0.150
Class Aa	83 (48.26%)	21 (61.76%)	
Non class Aa	89 (51.74%)	13 (38.24%)	
CPB time (min)	176.37 ± 65.32	178.47 ± 74.71	0.867
Aortic cross clamp time (min)	103.93 ± 24.03	111.35 ± 20.45	0.101
Circulatory arrest time (min)	27.55 ± 6.24	28.12 ± 6.43	0.631
Ventilator	105 (61.05%)	18 (52.94%)	0.379
Operative time (h)	10.68 ± 3.39	11.74 ± 4.42	0.050
Autologous blood ≥500 ml	61 (35.47%)	7 (20.59%)	0.092
Surgery type			0.946
AAR + TAR (TAVR) + FET	94 (54.65%)	18 (52.94%)	
Bentall + TAR (TAVR) + FET	40 (23.26%)	7 (20.59%)	
David + TAVR + FET	5 (2.91%)	1 (2.94%)	
Combine others	33 (19.19%)	8 (23.53%)	
RBCs (unit)	8.00 (5.50–12.00)	14.00 (10.12–20.50)	<0.001
Plasma (unit)	10.35 (6.50–15.22)	19.25 (14.72–28.15)	<0.001
Plasma/RBC ratio	1.41 ± 0.59	1.50 ± 0.66	0.417
Platelet (therapeutic dose)	1.00 (1.00–2.00)	2.00 (1.00–3.00)	0.010
Cryoprecipitate (therapeutic dose)	1.00 (1.00–2.00)	1.50 (1.00–2.75)	0.056
ICU stay (day)	6.00 (4.00–9.00)	9.50 (4.00–13.50)	<0.001

Results are expressed as *n* (%) or mean ± SD or medians (Q1–Q3). INR, international normalized ratio; APTT, activated partial thromboplastin time; PT, prothrombin time; FDP, fibrin/fibrinogen degradation product; CPB, cardiopulmonary bypass; AAR, ascending aorta replacement; TAR, total arch replacement; TAVR, total aortic vascular replacement; FET, frozen elephant trunk; RBCs, red blood cells; ICU, intensive care unit.

### 3.3. Univariate Cox regression analysis of in-hospital mortality

The results of the univariate Cox regression analysis associated with in-hospital mortality were shown in Table 2. In the univariate analysis, cerebrovascular disease (HR = 4.62, 95% CI: 2.01–10.62), RBCs transfusion (HR = 1.07, 95% CI: 1.04–1.11), plasma transfusion (HR = 1.07, 95% CI: 1.05–1.10), platelet transfusion (HR = 1.30, 95% CI: 1.01–1.68), cryoprecipitate transfusion (HR = 1.28, 95% CI: 1.07–1.53) and ICU stay (HR = 1.04, 95% CI: 1.02–1.07) were positively associated with the risk of in-hospital mortality. Other variables were not found to be significantly associated with in-hospital mortality in patients with AAAD.

**Table 2:** Univariate Cox regression analysis of in-hospital mortality.

Variables	In-hospital mortality (HR, 95% CI)	*P*-value
Age (years)	1.01 (0.98, 1.05)	0.381
Gender (male)	1.00 (0.50, 2.03)	0.993
INR	0.66 (0.10, 4.31)	0.665
APTT (s)	0.98 (0.93, 1.04)	0.511
PT (s)	1.01 (0.85, 1.19)	0.948
Leukocyte (10^9^/L)	1.00 (0.91, 1.08)	0.917
Platelet count (10^9^/L)	1.00 (0.99, 1.00)	0.363
Hemoglobin (g/L)	1.00 (0.98, 1.02)	0.762
D-dimer (μg/ml)	0.81 (0.58, 1.12)	0.206
FDP (μg/ml)	1.01 (0.97, 1.05)	0.628
Smoking	0.94 (0.47, 1.86)	0.857
Drinking	0.47 (0.21, 1.09)	0.078
Marfan syndrome	0.84 (0.20, 3.53)	0.817
Hypertension	0.71 (0.36, 1.43)	0.341
Diabetes mellitus	0.00 (0.00, Inf)	0.996
Hemopericardium	0.63 (0.15, 2.65)	0.533
Chronic renal failure	1.69 (0.41, 7.06)	0.471
Cerebrovascular disease	4.62 (2.01, 10.62)	<0.001*
Coronary artery disease	1.17 (0.41, 3.32)	0.770
**Penn class**		
Class Aa	1.0	
Non class Aa	0.59 (0.30, 1.18)	0.136
CPB time (min)	1.00 (1.00, 1.01)	0.798
Aortic cross clamp time (min)	1.01 (1.00, 1.03)	0.078
Circulatory arrest time (min)	1.01 (0.96, 1.07)	0.596
Ventilator	0.74 (0.38, 1.46)	0.391
Operative time (h)	1.06 (0.98, 1.13)	0.141
Autologous blood ≥500 ml	0.49 (0.21, 1.13)	0.093
**Surgery type**		
AAR + TAR (TAVR) + FET	1.0	
Bentall + TAR (TAVR) + FET	0.91 (0.38, 2.19)	0.841
David + TAVR + FET	1.13 (0.15, 8.46)	0.906
Combine others	1.20 (0.52, 2.76)	0.668
RBCs (unit)	1.07 (1.04, 1.11)	<0.001*
Plasma (unit)	1.07 (1.05, 1.10)	<0.001*
Plasma/RBC ratio	1.27 (0.74, 2.18)	0.385
Platelet (therapeutic dose)	1.30 (1.01, 1.68)	0.043*
Cryoprecipitate (therapeutic dose)	1.28 (1.07, 1.53)	0.008*
ICU stay (day)	1.04 (1.02, 1.07)	0.001*

HR, hazard ratio; CI, confidence interval. INR, international normalized ratio; APTT, activated partial thromboplastin time; PT, prothrombin time; FDP, fibrin/fibrinogen degradation product; CPB, cardiopulmonary bypass; AAR, ascending aorta replacement; TAR, total arch replacement; TAVR, total aortic vascular replacement; FET, frozen elephant trunk; RBCs, red blood cells; ICU, intensive care unit.

^*^ Indicates *P*-value < 0.05.

### 3.4. Multivariate Cox regression model for RBCs and plasma transfusion associated with in-hospital mortality

Multivariate Cox regression analysis was used to further evaluate the association between RBCs or plasma transfusion and the risk of in-hospital mortality in AAAD patients and the results were shown in Table 3. In the multivariable analysis, RBCs transfusion (HR = 1.08, 95% CI: 1.02–1.14) and plasma transfusion (HR = 1.09, 95% CI: 1.05–1.13) were independent risk factors for in-hospital mortality in Model I. After adjusted age and gender, we observed the HR was 1.02 and 95% CI was 0.97–1.07 for RBCs transfusion, HR was 1.07 and 95% CI was 1.03–1.11 for plasma transfusion. After adjusted age, gender, drinking, cerebrovascular disease, autologous blood ≥500 ml, ICU stay, operative time, platelet and cryoprecipitate, the similar results were observed in Model III (HR = 1.03 and 95% CI: 0.96–1.11 for RBCs transfusion, HR = 1.08 and 95% CI: 1.03–1.13 for plasma transfusion).

**Table 3:** Multivariate Cox regression model for RBC and plasma transfusion associated with in-hospital mortality.

Exposure	Model I (HR, 95% CI)	Model II (HR, 95% CI)	Model III (HR, 95% CI)
RBCs (unit)	1.08 (1.02, 1.14)*	1.02 (0.97, 1.07)	1.03 (0.96, 1.11)
Plasma (unit)	1.09 (1.05, 1.13)*	1.07 (1.03, 1.11)*	1.08 (1.03, 1.13)*

Model I, we adjusted none. Model II, we adjusted age and gender. Model III, we adjusted age, gender, drinking, cerebrovascular disease, autologous blood ≥500 ml, ICU stay, operative time, platelet and cryoprecipitate. HR, hazard ratio; CI, confidence interval.

^*^ Indicates *P*-value < 0.05.

### 3.5. Association between plasma/RBCs transfusion ratio and in-hospital mortality

**Figure 2:**
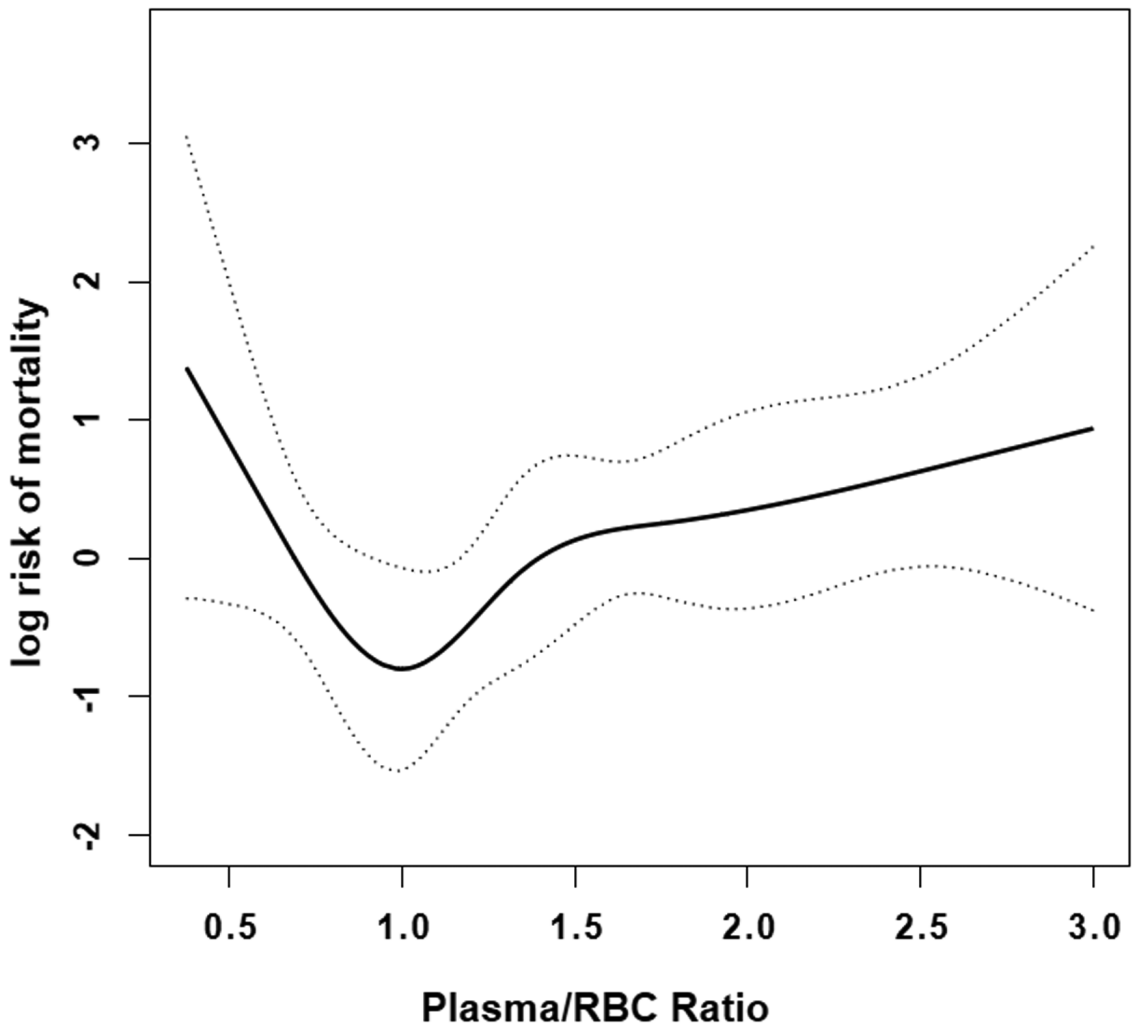
Smooth curve fitting curve. Nonlinear relationships between in-hospital mortality and plasma/RBCs transfusion ratio were observed after adjusting for covariates age, gender, drinking, cerebrovascular disease, autologous blood ≥500 ml, ICU stay, operative time, platelet and cryoprecipitate. Solid lines represent the spline plots and dotted lines represent the 95% CI of the spline plots.

After adjusted age, gender, drinking, cerebrovascular disease, autologous blood ≥500 ml, ICU stay, operative time, platelet and cryoprecipitate, we observed a nonlinear relationship between the plasma/RBCs transfusion ratio and the risk of death in AAAD patients (Figure 2). In the spline smoothing plot, mortality risk increased with plasma/RBCs transfusion ratio leveling up to the turning point 1:1. Optimal plasma/RBCs transfusion ratio with least mortality risk was 1. When the plasma/RBCs ratio was <1 (adjusted HR per 0.1 ratio: 0.28, 95% CI per 0.1 ratio: 0.17–0.45), mortality risk decreased with the increase of the ratio. When the plasma/RBCs ratio was 1–1.5 (adjusted HR per 0.1 ratio: 2.73, 95% CI per 0.1 ratio: 1.13–6.62), mortality risk increased rapidly with the increase of the ratio. When the plasma/RBCs ratio was >1.5 (adjusted HR per 0.1 ratio: 1.09, 95% CI per 0.1 ratio: 0.97–1.23), mortality risk tended to reach saturation, and increased non-significantly with increase of the ratio (Figure 2 and Table 4).

**Table 4:** Threshold analysis for the relationship between plasma/RBC transfusion ratio (per 0.1) and in-hospital mortality in patients with AAAD.

Models	Adjusted HR (95%CI)	LRT test (*P*-value)
Plasma/RBCs ratio turning point (1, 1.5)		0.025
<1 slope 1	0.28 (0.17, 0.45)*	
1–1.5 slope 2	2.73 (1.13, 6.62)*	
>1.5 slope 3	1.09 (0.97, 1.23)	

LRT test, Logarithmic likelihood ratio test (*P* < 0.05 indicates a non-linear relationship). Adjusted for covariates age, gender, drinking, cerebrovascular disease, autologous blood ≥500 ml, ICU stay, operative time, platelet and cryoprecipitate.

^*^ Indicates *P*-value < 0.05.

## 4. Discussion

In this study, we explored the relationship between plasma/RBCs ratio and in-hospital mortality in patients with AAAD. The results suggested that the optimal plasma/RBCs transfusion ratio related with least mortality risk was 1:1. Data from the Pragmatic, Randomized Optimal Platelet and Plasma Ratios (PROPPR) trial supported that early use of a 1:1 transfusion ratio was benefit for patients with rapid bleeding (22). In that trial, they found the early availability of transfusion using a plasma/RBCs ratio of 1:1 was effective in achieving hemostasis and reducing bleeding-related deaths. Besides the PRORRR trial, numerous non-randomized prospective and retrospective cohort studies explored the relationship between the transfusion ratios of different blood products and mortality. The results of these studies showed higher plasma/RBCs ratios were favorable (23–25). However, other researchers had found different results. Scalea TM et al. found there was no difference in mortality rates for the different plasma/RBCs ratios (26). Some researchers found high plasma transfusion or high plasma/RBCs ratios transfusion was associated with developing multi-organ failure, acute kidney injury and infectious complications (13, 27). Recent studies increasingly suggested that the use of transfusion ratios closed to whole blood was beneficial in the early phases of resuscitation (28). Based on the idea of restoring the normal hemostatic balance of procoagulant and anticoagulation as soon as possible, experts recommended a 1:1 plasma/RBCs transfusion ratio. These recommendations were echoed in a clinical practice guideline developed by US combat hospitals in September 2004 (29). And we also found a 1:1 plasma/RBCs ratio closed to whole blood was the optimal plasma/RBCs ratio. It indicated that maintaining the appropriate ratio was beneficial for the AAAD patients.

Aortic dissection could cause blood to flow through a non-endothelialized false lumen. Blood contacted with inner subcutaneous tissue factors, collagen, and the outer layer of the aortic wall. It leaded to consumable coagulopathy, which was manifested as a decrease in coagulation factors and a significant elevation in fibrin/fibrinogen degradation products (30). In addition, platelet counts and coagulation factors such as V, VII, VIII and XI could also decrease causing by cardiopulmonary bypass. While the fibrin/fibrinogen degradation products, plasmin-antiplasmin complex, and factor XIIa increased causing by cardiopulmonary bypass (31). Bleeding, coagulation factor dysfunction, platelet dysfunction and thrombocytopenia commonly occurred during the surgery. Based on the above events, plasma and RBCs were frequently transfused to provide coagulation factors, either as a preventive measure or as a treatment for hemorrhagic complications (32, 33). However, blood transfusion could increase morbidity and mortality of patients underwent cardiac surgeries (34), but there were few studies on the relationship between blood transfusion and hospital mortality in AAAD patients. We found plasma transfusion was an independent risk factor of in-hospital mortality. Possibly due to the small sample size, the association between RBCs transfusion and the risk of in-hospital mortality was not significant in the adjusted model, but the effect value indicated a 3% increase in mortality risk for each additional unit of RBCs transfusion. In Bjursten et al.'s study, they found no association between RBCs transfusion and mortality, whereas plasma transfusion was associated with increased mortality (35). In Murad MH et al.'s single retrospective study, they found there was an association between plasma transfusion and lower mortality in intracranial hemorrhage patients. While in surgery patients without massive transfusion, an increased mortality associated with plasma transfusion was observed (36). A previous study found that the degree of reduced survival rate of cardiac surgical patients was related with the number of RBCs transfused (37). Therefore, optimal dose, timing and ratio of plasma and/to RBCs transfusion were important.

The strength of this study was to visualize the relationship between plasma/RBCs transfusion ratio and in-hospital mortality in patients with AAAD. Previous studies divided plasma/RBCs transfusion ratio into 1:1, 1:2, 1:4, etc. The plasma/RBCs transfusion ratio was analyzed as a continuous variable in this study. In this way, we could observe the death risk of AAAD patients corresponding to each plasma/RBCs transfusion ratio from the fitted curve.

There were still some limitations that should be noted. First, this study was a retrospective and single-center design, and it was uncertain to what extent these results could be generalized to all patients, or other centers that use different surgical techniques or blood conservation strategies. Despite this limitation, the study suggested a benefit of optimal plasma/RBCs ratio (1:1) for in-hospital mortality with AAAD patients and further affirmed the plasma/RBCs ratio (1:1) recommended by experts (29). Second, the ratios of platelet and cryoprecipitate transfusion were not considered in our analysis. However, we adjusted platelet and cryoprecipitate to minimize the bias caused by these two variables in this analysis. Third, the information of thrombelastography (TEG) could describe the coagulopathy of patients, but due to lack of data, we didn't include TEG in this study. However, variables PT, APTT, INR, D-dimer and FDP partially compensated for this limitation.

## 5. Conclusions

In conclusion, our study indicated that a 1:1 plasma/RBCs ratio was associated with the lowest mortality in the patients with AAAD. And there was non-linear relationship existed between plasma/RBCs transfusion ratio and in-hospital mortality. Optimal dose of RBCs or plasma transfusion and suitable RBCs/plasma transfusion ratio could reduce mortality risk in patients with AAAD. It is very important for clinical blood transfusion, especially for patients with a large volume of blood transfusions.

## Data Availability

The raw data supporting the conclusions of this article will be made available by the authors, without undue reservation.
